# Advances in Nanotechnological Strategies for Preserving and Authenticating Bioactive Compounds in Extra Virgin Olive Oil: Nano-Enabled Stabilization, Sensing, and Circular Valorization

**DOI:** 10.3390/foods15081278

**Published:** 2026-04-08

**Authors:** José Roberto Vega Baudrit, Yendry Corrales-Ureña, Karla Jaimes Merazzo, Javier Stuardo Chinchilla Orrego, Mary Lopretti

**Affiliations:** 1 Laboratorio Nacional de Nanotecnología LANOTEC CENAT CONARE, San José 1174-1200, Costa Rica; ycorrales@cenat.ac.cr (Y.C.-U.); javier.chinchillaorrego@outlook.com (J.S.C.O.); 2 Escuela de Química, Universidad Nacional, Heredia 40101, Costa Rica; 3 SINTEF Helgeland AS, Halvor Heyerdahls vei 33, 8626 Mo i Rana, Norway; karla.merazzo@sintef.no; 4 Materials Science and Engineering Research Center (CICIMA), University of Costa Rica, San Pedro 11501-2060, Costa Rica; 5 Department of Nuclear Techniques in Biochemistry and Biotechnology, Center for Nuclear Research, Faculty of Sciences, University of the Republic (UdelaR), Montevideo 11400, Uruguay; mlopretti@gmail.com

**Keywords:** extra virgin olive oil, phenolic compounds, oxidative stability, nanoencapsulation, Pickering emulsions, authenticity, food fraud, sensors, traceability, circular biorefinery

## Abstract

Extra-virgin olive oil (EVOO) is a chemically complex lipid matrix whose minor constituents—especially phenolic secoiridoids—drive sensory quality, oxidative stability, and health benefits. However, these bioactives are vulnerable to heat, light, oxygen, and pro-oxidant metals during processing and distribution, while the high cost of EVOO often makes it a target for adulteration and mislabeling. This review critically assesses nano-enabled, food-grade strategies that (i) preserve phenolics and aroma compounds through nanoencapsulation, inclusion complexes, Pickering stabilization, and structured lipid systems; (ii) control their release and bioaccessibility during digestion; and (iii) enhance authenticity verification via sensor-ready packaging, spectroscopy/chemometrics, and digital traceability systems (IoT, machine learning, blockchain). We align these innovations with the “product identity constraints” of the EVOO category and with official quality standards used in routine control (IOC/EU). Finally, we explore circular valorization of olive-mill by-products within food-centered biorefineries, outlining pathways to convert biomass into ingredients, materials, and energy, thus reducing environmental impacts. Research priorities are proposed to develop scalable, regulation-compliant nanotechnologies that extend shelf life and increase consumer trust without compromising EVOO category standards.

## 1. Introduction

Extra virgin olive oil (EVOO) is a hallmark component of the Mediterranean dietary pattern and a premium lipid food whose value depends on a favorable fatty-acid profile and a chemically rich minor fraction. Phenolic compounds—including hydroxytyrosol and tyrosol derivatives, secoiridoids, lignans, and flavonoids—shape bitterness, pungency, oxidative stability, and many of the health-related properties attributed to EVOO [1,2,3,4,5,6,7,8,9,10,11,12]. Recent reviews continue to support the relevance of EVOO phenolics to cardiometabolic protection and to the EFSA framework linking olive-oil polyphenols with protection of LDL particles from oxidative damage [5,6,7,8,9,10,12].

At the same time, the bioactive fraction is highly variable and chemically labile. Cultivar, ripening stage, processing conditions, packaging, headspace oxygen, light, temperature, and storage time all influence phenolic retention, volatile evolution, and oxidative indices [13,14,15,16,17,18,19,20,21,22,23,24,25,26,27,28,29,30]. These coupled degradation pathways complicate shelf-life prediction and strengthen the need for preservation strategies that remain compatible with official EVOO quality criteria.

### Olive Production Continuum and Product Identity

**Figure 1:**
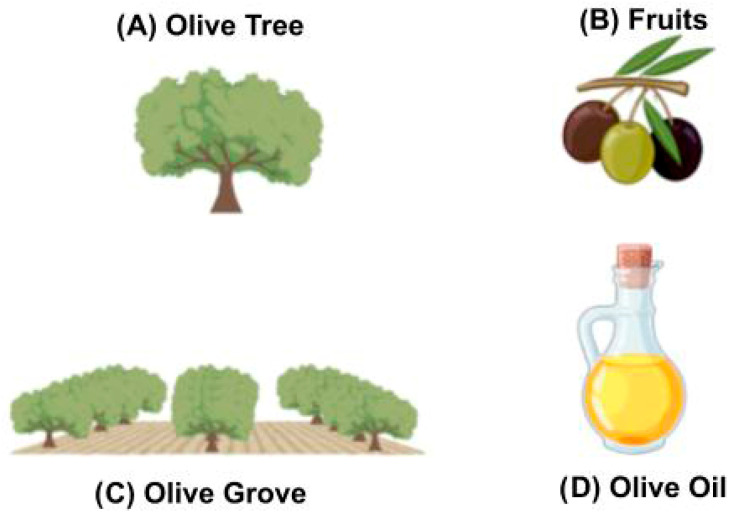
Integrated representation of the olive production system. (**A**) Whole olive tree (*Olea europaea* L.) showing trunk, branching architecture, and foliage; (**B**) Olive fruits at different ripening stages, including green (unripe), turning (veraison), and fully ripe (black); (**C**) Olive grove illustrating cultivated landscape structure and spatial tree distribution; (**D**) Olive oil as the final product derived from fruit processing. The figure conceptually links plant morphology, fruit development, agricultural context, and value-added product within a single production continuum.

Figure 1 summarizes the biological and agro-industrial continuum that connects olive-tree architecture, fruit ripening, orchard context, and the final oil. This systems view is useful because the technological questions addressed in this review—stability, authenticity, and circular valorization—originate upstream in cultivar choice, harvest timing, agronomic management, and post-harvest handling.

Figure 1A highlights canopy architecture as a functional trait linked to light interception, aeration, pruning, and harvesting efficiency.

Figure 1B condenses the transition from green to ripe fruit. These stages coincide with changes in chlorophylls, phenolics, and lipid accumulation that directly affect yield, oxidative stability, and sensory quality [13,14,15,16,17,18,19,20,21,22,23,24,25,26,27,28,29].

Figure 1C places olive production at the agroecosystem level, where planting density, soil management, water availability, and landscape configuration influence both productivity and environmental performance.

Figure 1D links fruit physiology to EVOO composition. Mechanical extraction preserves the minor bioactive constituents that help define nutritional quality, sensory character, and commercial value [1,2,3,4,5,6,7,8,9,10,13,14].

Together, these panels clarify why EVOO preservation and authentication cannot be separated from agronomy, processing, and sustainability.

EVOO is also a frequent target of adulteration and origin misrepresentation because of its premium price. Authentication, therefore, requires multidimensional evidence, including compositional markers, process history, and increasingly traceability data [31,32].

Nanotechnology and nano-enabled systems (broadly defined here as colloidal or structured systems with critical features at the nanoscale) offer a convergent pathway to address these pressures. In the EVOO context, nano-strategies can be positioned in three complementary roles:

1. Preservation: protection of oxidation-sensitive bioactives and aroma markers through physical barrier effects, partition control, and controlled release [33,34,35,36,37,38,39,40,41,42,43,44,45,46,47,48,49,50,51,52,53,54,55,56,57,58,59].
2. Nutrition and function: enhancement of bioaccessibility and targeted release in digestion-relevant environments [60,61,62,63].
3. Trust and traceability: integration of sensors, chemometrics, and digital infrastructures to improve authenticity screening and track-and-trace performance [31,32,64,65,66,67,68,69,70,71,72,73,74,75,76,77,78,79,80,81,82].

Accordingly, this review focuses on mechanism-informed preservation, realistic analytical and authentication workflows, digital traceability, and circular biorefinery routes that remain compatible with EVOO product identity and food regulation [82,83].

## 2. Methodology of the Review

This article was prepared as a structured narrative review informed by systematic literature mapping rather than as a formal systematic review or meta-analysis. This format was selected because the topic spans EVOO chemistry, oxidation mechanisms, nano-enabled stabilization, authentication analytics, smart packaging, and circular valorization. The evidence base is therefore interdisciplinary and methodologically heterogeneous.

Literature identification combined iterative searches in major bibliographic databases with manual screening of references from key reviews, standards, and primary studies. For this revised version, the search was refreshed through February 2026, with targeted emphasis on 2020–2026 publications addressing packaging-dependent stability, food-grade nanoformulations, deployable sensing, chemometric authentication, digital traceability, and olive by-product valorization. Search terms combined EVOO-specific descriptors (e.g., EVOO, olive phenolics, hydroxytyrosol, tyrosol, secoiridoids, oleuropein) with technology- and application-oriented terms such as nanoencapsulation, nanoemulsion, Pickering emulsion, nanostructured lipid carrier, biopolymer nanoparticles, cyclodextrin inclusion complexes, organogels, oxidative stability, shelf life, packaging, authenticity, spectroscopy, chemometrics, metabolomics, sensor technologies, traceability, machine learning, blockchain, olive pomace, olive mill wastewater, circular economy, and biorefinery.

Seminal publications on EVOO chemistry, phenolic composition, and regulatory frameworks were retained when they remain foundational. However, greater weight was assigned in the synthesis to recent literature on translational performance, scale-up, validation in real matrices, and current regulatory requirements. Inclusion was primarily limited to peer-reviewed scientific articles, regulatory or standard-setting documents (including EFSA guidance, International Olive Council standards, and relevant European Union legislation), and a limited number of high-quality reviews used to contextualize the field and identify additional primary sources.

Priority was given to studies directly addressing EVOO, olive-derived phenolics, or food-grade lipid systems with clear mechanistic or technological relevance. Evidence from related edible-oil systems or plant phenolic matrices was included only when it provided transferable insights into stabilization mechanisms, formulation strategies, analytical workflows, or authenticity assessment relevant to EVOO. Studies that focused exclusively on pharmaceutical nanomaterials and lacked plausible transferability to edible systems were not considered.

For each eligible source, the analysis captured formulation variables, physicochemical descriptors, storage or digestion conditions, analytical endpoints, and any evidence relevant to scalability, regulatory feasibility, or validation in real oil matrices. Because the literature is heterogeneous in design and endpoints, a quantitative meta-analysis was not appropriate. Instead, the evidence was synthesized qualitatively, with emphasis on mechanistic clarity, robustness of controls, completeness of characterization, industrial relevance, and compatibility with EVOO identity requirements. Claims related to digestion or bioaccessibility were interpreted preferentially when supported by standardized in vitro digestion methodologies.

## 3. Bioactive Compounds in EVOO and Major Degradation Drivers

### 3.1. Phenolic Profile and Functional Relevance

**Figure 2:**
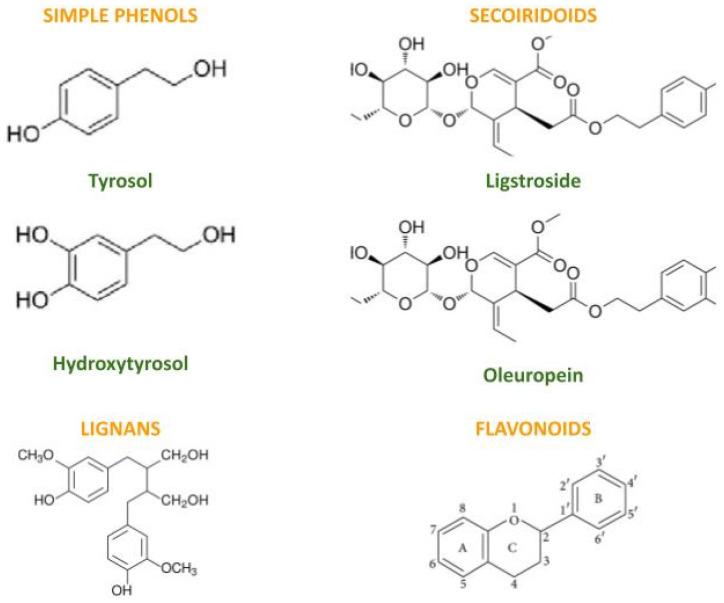
Structural phenolic profile of EVOO systems. In the flavonoid scaffold, the letters A, B, and C denote the three rings of the molecule, while the numbers indicate the conventional numbering of carbon atoms and substitution positions. The primed numbers (e.g., 2′, 3′, 4′) correspond specifically to the B ring, allowing precise identification of hydroxylation or other functional group patterns.

EVOO phenolics are commonly partitioned into simple phenols (e.g., hydroxytyrosol, tyrosol), secoiridoid derivatives (e.g., oleuropein and ligstroside aglycones and related dialdehydic forms), lignans, and flavonoids (Figure 2) [1,2,3,4,13,14,15,19]. Their extraction and quantification have historically relied on HPLC-based methods and refined procedures for isolating polar fractions [13,14,21]. Analytical improvements (e.g., APCI-MS and LC-SPE-NMR) have enhanced the structural resolution of complex secoiridoids and minor phenolics [22,23]. From a functional standpoint, the antioxidant behavior of EVOO phenolics extends beyond radical scavenging to include interactions with tocopherol regeneration and modulation of oxidative chain reactions, which is particularly relevant in lipid matrices [53].

### 3.2. Oxidation Pathways in EVOO

**Figure 3:**
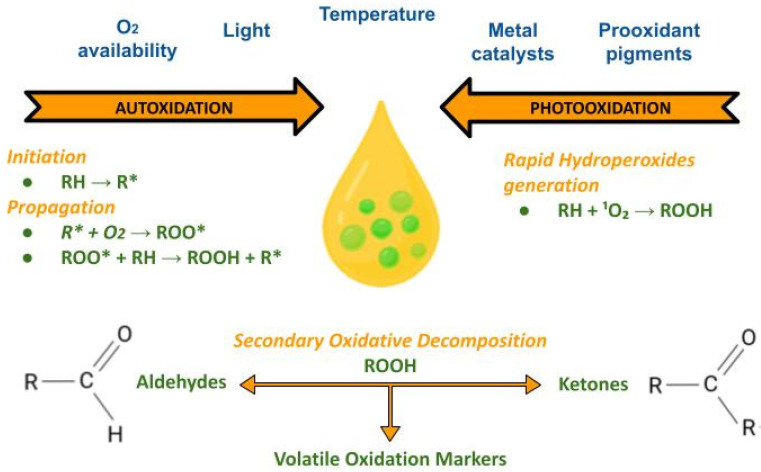
Simplified scheme of the main oxidation routes relevant to EVOO stability, highlighting how oxygen, light, temperature, and metal catalysts contribute to hydroperoxide formation and secondary volatile generation (The symbol “*” in the figure represents a **free radical**, meaning a chemical species with an **unpaired electron**).

Oxidative degradation in edible oils is driven by a network of reactions influenced by oxygen availability, light, temperature, metal catalysts, and prooxidant pigments (Figure 3). In EVOO, photooxidation is particularly relevant because chlorophyll derivatives can sensitize the formation of singlet oxygen, thereby accelerating hydroperoxide generation [24]. Autoxidation leads to the formation of hydroperoxides, which subsequently decompose into aldehydes, ketones, and other volatile markers that contribute to sensory defects such as rancidity [17,18,30]. The interplay between oxidative indices and volatile signatures is central to both shelf-life assessment and authentication (since compositional changes can mimic adulteration signals if not properly modeled) [29,30,53].

### 3.3. Packaging, Headspace, and Storage as Controllable Determinants

Packaging remains one of the most controllable levers in industry. Studies comparing packaging materials highlight the influence of oxygen permeability and light exposure on chemical indices and sensory attributes [25,26,27,28]. Headspace oxygen fraction and its dynamic exchange with permeable packaging materials further modulate oxidation kinetics during storage. In this context, controlled reductions in headspace oxygen (e.g., 2–5%) have been shown to significantly delay oxidative deterioration and extend shelf life in EVOO under both clear and dark storage conditions [84]. The presence of oxygen scavengers in polymer packaging can further attenuate oxidative trajectories under certain storage regimes [25]. Bag-in-box formats have been evaluated as alternatives to traditional glass packaging, with performance contingent on oxygen ingress, headspace management, and handling behavior [28]. Predictive shelf-life models underscore that degradation is not purely time-dependent but is driven by a set of coupled variables (temperature, oxygen availability, phenolic reserve, and initial quality state) [29].

## 4. Nanotechnological Strategies for Bioactive Preservation and Delivery

### 4.1. Rationale and Mechanism: Why Nanoscale Matters

From a physical chemistry standpoint, nano-enabled systems can stabilize bioactives by:

(i) reducing interfacial area exposure to oxygen or light through engineered barriers;
(ii) controlling the partitioning of phenolics between polar domains and lipid phases;
(iii) modulating local redox microenvironments and diffusion pathways; and
(iv) enabling controlled release or targeted bioaccessibility in digestion models [53,54,55,56,57,58,59,60,61,62,63].

**Figure 4:**
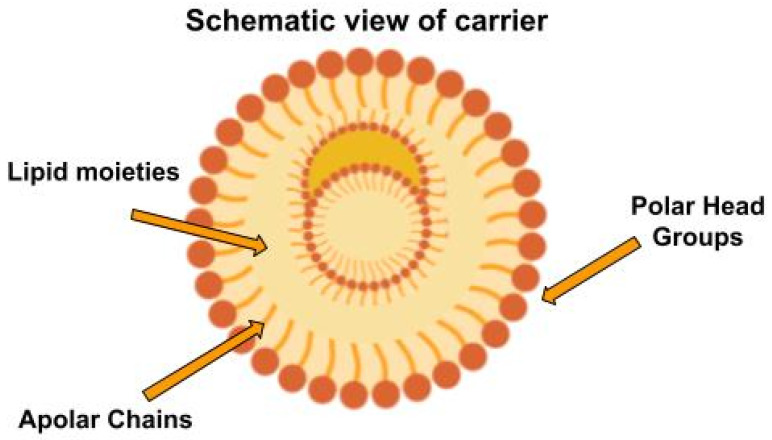
Schematic representation of the internal distribution of lipid moieties within nanostructured lipid carrier droplets at increasing solid-to-liquid lipid fractions. Original schematic based on the molecular-dynamics trends reported in [85].

Figure 4 provides a schematic representation of the internal distribution of lipid moieties within nanostructured lipid carrier droplets at increasing solid-to-liquid lipid fractions. EVOO contains a complex pool of minor bioactive compounds, including phenolic secoiridoids, simple phenols, tocopherols, and squalene, which are highly susceptible to oxidative degradation during processing, storage, and gastrointestinal digestion. Environmental and technological stressors, such as oxygen, light, temperature, and pro-oxidant catalysts, activate photooxidative and autoxidative pathways, leading to phenolic depletion, the formation of volatile off-flavors, and reduced bioaccessibility. Nanotechnological intervention strategies—including nanoemulsions, lipid and polymeric nanoparticles, Pickering emulsions, organogels, and cyclodextrin complexes—act as protective and controlled-release systems, limiting degradation and enhancing functional outcomes such as oxidative stability, bioactive preservation, improved gastrointestinal delivery, and extended shelf life.

### 4.2. Lipid-Based Nanocarriers: NLCs and Related Systems

Lipid nanoparticles are attractive for lipophilic or amphiphilic bioactives because they offer food-compatible lipid matrices and high encapsulation efficiency. A representative example is the encapsulation of oleuropein (OLE) in nanostructured lipid carriers (NLCs), with demonstrated cellular compatibility and preserved antioxidant efficacy in a relevant epithelial model. NLCs are based on the formation of a partially disordered lipid matrix at the nanoscale, composed of both a solid and a liquid lipid phase. This structural configuration increases loading capacity, reduces expulsion during storage, enables sustained release, and enhances antioxidant performance compared to the free compound [33]. Such systems can be interpreted as “structured micro-reservoirs” that protect labile phenolics from environmental exposure while preserving functionality at the point of delivery. The release profile can be tailored by adjusting the solid-to-liquid lipid ratio and the internal crystallinity of the lipid matrix, thereby controlling the diffusion of encapsulated phenolic compounds such as OLE [33]. This is particularly relevant for phenolics in olive-derived systems, whose antioxidant efficacy depends on both stability and bioavailability.

The relevance of this approach to EVOO systems is strongly supported by the well-established mechanisms of lipid oxidation. Edible oil oxidation occurs primarily through radical-mediated autoxidation and singlet oxygen-mediated photooxidation, leading to hydroperoxide formation and subsequent degradation products that compromise nutritional and sensory quality [53]. Although EVOO exhibits relatively high oxidative stability due to its monounsaturated fatty acid profile and natural antioxidants, oxidative degradation remains a critical challenge during processing and storage. Therefore, strategies aimed at stabilizing or enhancing the functional activity of endogenous phenolic compounds are directly aligned with the mitigation of oxidation pathways [53].

A key design constraint is oxidative compatibility; the carrier itself must not introduce prooxidant lipids or surfactants that accelerate degradation. This necessitates selecting carriers and antioxidant co-stabilization strategies that align with the mechanisms of oxidative degradation in edible oils [53].

From a mechanistic perspective, ref. [85] investigated the molecular self-assembly process that occurs during nanoparticle formation. The organization of lipid molecules is primarily governed by hydrophobic interactions among hydrocarbon chains and by the preferential orientation of polar head groups toward the aqueous interface. In the simulations presented (see Figure 4), the polar head groups of the lipids are shown to orient toward the nanoparticle’s outer region, forming a surfactant-stabilized interface, while the apolar chains compact within the nanoparticle’s inner core. This organization is not perfectly crystalline; rather, the mixture of lipids with different melting points introduces structural disorder, generating microdomains with distinct density and polarity [85].

Regarding the localization of the encapsulated molecule within the NLC, molecular dynamics simulations indicate that the final position of the bioactive compound depends on its relative polarity and its ability to establish specific interactions with the lipid components. Highly hydrophobic molecules tend to insert deeply into the lipid core, stabilized by van der Waals interactions with the hydrocarbon chains. In contrast, molecules with partially polar regions may be located at interfacial zones, interacting both with the polar head groups and the surrounding aqueous environment. This finding is critical for understanding how the encapsulated molecule is structurally accommodated within the carrier and how its spatial distribution influences its release profile [85].

Moreover, the global morphology of the nanocarrier can be modulated by changes in lipid composition. Variations in the solid-to-liquid lipid ratio alter the core density and the degree of molecular ordering, directly affecting the thermodynamic stability of the system. From a mechanistic standpoint, this implies that encapsulation is not merely a physical entrapment phenomenon, but rather a dynamic process governed by the balance of intermolecular forces, structural reorganization, and minimization of the system’s free energy. The simulations further demonstrate that the presence of the bioactive compound may induce slight perturbations in lipid packing, confirming a bidirectional interaction: the matrix organizes the compound, while the compound also influences the matrix architecture [85].

Finally, ref. [85] emphasizes that the internal structure of NLCs is dynamic rather than static. Molecular dynamics trajectories reveal continuous fluctuations in lipid chain orientation and in the mobility of the encapsulated compound. This molecular mobility mechanistically underlies diffusion and controlled-release processes. The encapsulated molecule does not remain immobile within the core; instead, it undergoes microdisplacements within lipid domains whose stability depends on temperature, composition, and chemical environment [85].

This level of structural and dynamic description is particularly relevant for applications in complex systems such as EVOO, where preserving phenolic compounds requires not only efficient encapsulation but also a deep understanding of molecular interactions within the lipid-based system.

Beyond the nanocarrier’s internal conformation, molecular simulations of NLCs have enabled direct investigation of interactions between bioactive molecules and lipid components. For instance, studies combining molecular docking and molecular dynamics have developed three-dimensional NLC models formulated with lipids derived from natural sources and have analyzed how the encapsulated molecule accommodates within the matrix and remains stable following computational self-assembly [86]. These analyses reveal not only the spatial distribution of bioactives within the carrier but also how specific interactions (e.g., van der Waals forces and hydrophobic interactions) determine encapsulation stability and efficiency, thereby underpinning the molecular delivery mechanisms described in the specialized literature [86].

Additionally, in EVOO systems, these technologies can be conceptually linked to oxidation control mechanisms for quality control purposes. Since lipid oxidation generates specific primary and secondary products (e.g., hydroperoxides and aldehydes) [53], understanding these pathways enables the design of packaging strategies that monitor oxidative status. Although the cited studies do not experimentally evaluate sensor-integrated packaging, mechanistic knowledge of oxidation chemistry provides the scientific basis for developing sensor-ready systems that detect oxidation markers. In parallel, nanoencapsulation strategies, such as NLCs, may help preserve phenolic fingerprints and oxidative stability, thereby indirectly supporting authenticity through controlled antioxidant protection [33].

In summary, NLCs provide a structurally tunable lipid matrix that can enhance phenolic stability, modulate release kinetics, and potentially improve bioaccessibility, while lipid oxidation mechanisms in edible oils establish the chemical framework necessary to justify both stabilization strategies and the development of monitoring systems for quality and authenticity assurance. However, their lipid components may themselves be prone to oxidation if not carefully selected. Surfactant choice is critical, as it can influence oxidative stability and long-term structural integrity.

### 4.3. Polymeric and Biopolymer Nanoparticles

Preserving EVOO phenolics is essential for maintaining functional and sensory quality. Oleuropein and hydroxytyrosol are especially relevant because they contribute to antioxidant activity and oxidative stability [2,87]. Yet these molecules are chemically labile. Polymeric nanoparticles, including biodegradable PLA-based systems, therefore attract attention as protective carriers that can provide structural robustness and controlled release [35].

Oleuropein is amphiphilic because it contains multiple hydroxyl groups and an ester functionality. Hydroxytyrosol is smaller and is a strong hydrogen donor [2,87]. These structural differences influence how each molecule interacts with biopolymeric environments. Protein–phenol model studies consistently show non-covalent binding through hydrogen bonding and hydrophobic interactions [88,89].

In β-lactoglobulin systems, tea polyphenols bind spontaneously and occupy energetically favorable hydrophobic regions. Spectroscopic and modeling data also show that binding can alter protein secondary structure [89].

Hydroxytyrosol exhibits behavior analogous to that of bovine serum albumin. Fluorescence quenching and docking studies identified a defined binding pocket stabilized by hydrogen bonds and hydrophobic contacts [88].

These results do not come from fully formed food nanoparticles. However, they clarify the intermolecular forces likely to govern phenolic organization in protein-based nanocarriers [88,89].

Polysaccharide-based evidence points in the same direction. Chitosan nanoparticles loaded with olive-pomace phenolics showed FTIR shifts consistent with hydrogen bonding between phenolic hydroxyl groups and protonated amines, with no evidence of covalent conjugation [90]. The formulation also retained antioxidant activity and improved stability relative to the free extract.

Two mechanistic questions are especially important. First, where is the molecule located? Available data suggest that phenolics preferentially occupy energetically favorable hydrophobic or interfacial domains rather than remaining randomly dispersed [88,89].

In polysaccharide-based nanoparticles, such as chitosan systems, phenolic compounds are associated with functional groups capable of forming hydrogen bonds and electrostatic interactions. The study reported FTIR spectral shifts consistent with non-covalent interactions between phenolic hydroxyl groups and protonated amine groups of chitosan, supporting supramolecular incorporation within the nanoparticulate matrix [90].

Second, how does the guest affect the carrier? Phenolic binding can reorganize the host matrix, as reflected by changes in protein secondary structure and supramolecular packing [88,89,90]. These reversible interactions may help shield phenolics from oxygen and other pro-oxidant species.

Beyond experimental characterization, computational methodologies provide additional structural insight. A review describes the application of molecular dynamics (MD) and coarse-grained simulations in polymer-based nanocarriers, demonstrating how these approaches enable visualization of polymer self-assembly, cargo localization, and interaction energies at molecular and mesoscale resolution [91]. Although their work focuses primarily on drug delivery systems, the principles described (particularly regarding the role of non-covalent interactions in determining cargo distribution within polymeric architectures) are mechanistically transferable to phenolic encapsulation systems. Such simulation frameworks offer a powerful tool for predicting molecular ordering and rationalizing experimental observations in polymeric nanoencapsulation strategies [91].

In practical formulations, polymeric nanoencapsulation relies on polymer self-assembly during transfer from an organic phase to an aqueous phase. Encapsulation efficiency depends on the choice of solvent, polymer–phenol affinity, and phase partitioning [35]. This translational route is promising, but it remains constrained by residual-solvent control, migration testing, digestion behavior, and scale-up costs.

While such approaches have strong translational logic, they must be critically evaluated for food-grade compatibility (monomer residues, solvent traces, and migration profiles) and for digestion behavior.

**Figure 5:**
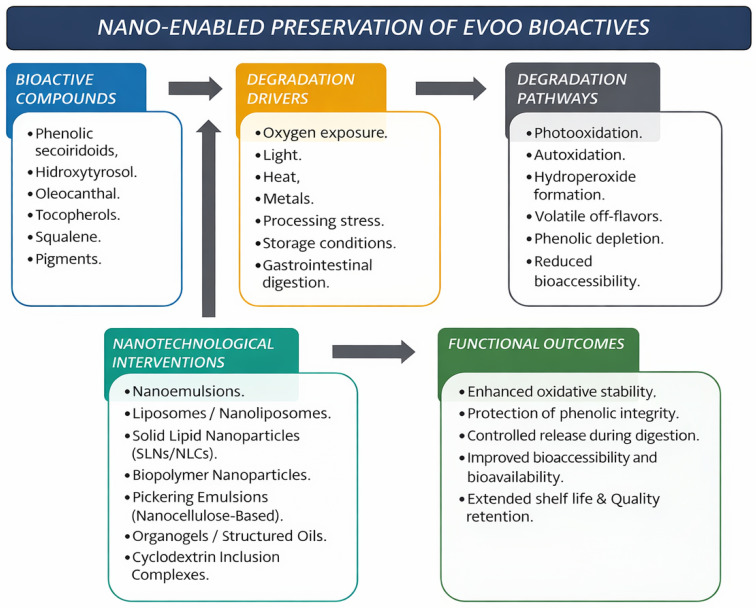
Conceptual framework for nano-enabled preservation of EVOO bioactives.

Figure 5 schematically links oxidative stressors, degradation routes, and representative nano-enabled interventions that can reduce diffusion, shield bioactives, and modulate release during gastrointestinal transit.

Biopolymeric nanoparticles and complexes (e.g., protein–polysaccharide systems) represent a further step toward food-native materials. Reviews of biopolymeric nanostructures emphasize their potential to protect phytochemicals under processing stress and to control release profiles [59]. The practical advantage in EVOO applications is that many such materials are already accepted as food ingredients, thereby reducing regulatory friction relative to novel engineered nanomaterials.

Beyond protection, nanostructured materials can also act as sensing platforms. Interactions between oxidation products and nanoelectronic surfaces may generate electrical, optical, or colorimetric signals, enabling real-time monitoring of oxidative drift or adulteration [59]. In principle, an EVOO-oriented system could couple phenolic preservation with sensor-ready packaging that reports changes in chemical status during storage and distribution.

### 4.4. Cyclodextrin Inclusion Complexes and Hybrid Nano-Reservoirs

Cyclodextrins provide a well-established inclusion mechanism for phenolics and aroma-active compounds. Encapsulation of olive leaf extract within β-cyclodextrin (β-CD) has been demonstrated, thereby improving stability and facilitating its incorporation into functional matrices [37]. Hybrid systems—combining cyclodextrins with polysaccharide nanoparticles—have been proposed to improve storage stability of olive oil components and mitigate oxidation under stress. Mechanistically, these systems can act via molecular encapsulation (inclusion), diffusion limitation, and microenvironmental modulation.

The methodological principle is based on β-CD’s ability to form inclusion complexes, as it is a cyclic molecule with a hydrophobic inner cavity and a hydrophilic outer surface. This architecture allows hydrophobic or partially hydrophobic molecules (such as OLE) to “enter” the cavity via host–guest interactions without forming permanent covalent bonds [37].

Complex formation was confirmed by DSC, in which the thermal transition peaks of the extract disappeared upon complexation. This indicates that the compound is no longer in its free state but is protected within the β-CD cavity, thereby enhancing its stability against thermal oxidation. Furthermore, the molecules retained within the β-CD complex exhibited improved aqueous solubility and stability, as they were protected from oxidative degradation. Together, these results provide a solid methodological basis for encapsulating sensitive bioactive compounds and incorporating them into EVOO production systems to reduce the direct exposure of phenolic compounds to oxygen, thereby reducing degradation reactions and preserving quality and authenticity standards [37].

Inclusion in β-cyclodextrin is a supramolecular process governed by steric complementarity and dispersive stabilization [92]. Molecular simulations show partial insertion of aromatic guests into the hydrophobic cavity, with polar groups oriented toward the cyclodextrin portals. Residence times are finite, indicating a dynamic rather than permanent complex. This behavior is relevant to aromatic EVOO phenolics.

Within the context of sensor-ready packaging, β-CD could also be integrated into packaging materials to selectively capture volatile compounds or phenolic markers, and to facilitate molecular release or exchange when environmental conditions change (temperature, humidity, or oxidative stress). In this framework, β-CD acts not only as a preservation system but also as a passive molecular transducer, whose reversible interactions can be incorporated into sensing platforms aimed at quality monitoring [37].

β-CD systems are constrained by molecular and formulation-level limitations. At the molecular scale, encapsulation efficiency is limited by cavity size compatibility and potential displacement by competing molecules in complex matrices such as EVOO. Additionally, β-CD does not inherently provide macroscopic protection against oxygen diffusion. When integrated into hybrid nanoparticulate systems, formulation complexity and production costs increase, as synergistic stabilization requires precise control of host–guest interactions and matrix compatibility. Moreover, multistep processing may complicate scale-up and industrial reproducibility, while dosage limits and economic considerations can further restrict large-scale implementation.

### 4.5. Nanoemulsions and Pickering Emulsions

Nanoemulsions provide small droplet size and flexible formulation design. However, they remain thermodynamically unstable systems, and surfactant choice can either improve or worsen oxidative stability [45,46].

Pickering emulsions use solid colloidal particles instead of molecular surfactants. When particle concentration, surface properties, pH, and ionic strength are controlled, the interfacial layer can reduce coalescence and slow oxidation [38,39,40,41,42,43,44]. The trade-off is formulation complexity, sensitivity to aggregation, and the need for careful particle sourcing and regulatory assessment.

In EVOO-based oil-in-water nanoemulsions, lipophilic compounds are located mainly within the droplet core, whereas amphiphilic surfactants organize a dynamic interfacial layer. Structural studies indicate that encapsulated molecules can increase interfacial ordering and local microviscosity, which helps explain improved kinetic stability and protection against oxidation [93].

Pickering emulsions stabilized by α-cyclodextrin follow a different mechanism. Oil molecules form host–guest inclusion complexes that self-assemble into interfacial particles, producing a rigid barrier around the droplets [94]. This shell can improve physical stability, but it also introduces formulation constraints related to particle growth, reproducibility, and process control.

Collectively, these studies highlight that interfacial molecular organization plays a central role in determining both the encapsulation mechanism and the physicochemical stability of bioactive compounds in emulsion-based delivery systems. Whereas nanoemulsions rely on the self-organization of surfactant molecules into a flexible interfacial monolayer, Pickering emulsions are stabilized by the irreversible adsorption of supramolecular particles derived from inclusion complexes, which form a solid protective shell around the oil droplets [93,94].

Recent work increasingly emphasizes the use of renewable stabilizers and digestive control [40,41,84]. Even so, these systems must still demonstrate robustness in real foods, compatibility with processing, and cost-effective manufacturing.

### 4.6. Organogels and Structured Oils

Structured organogels based on low-molecular-weight gelators represent a robust nanotechnological strategy for the preservation and controlled delivery of lipophilic compounds in oil-based matrices. Several studies have demonstrated that the thermally induced crystallization of compounds such as policosanol, glycerol monopalmitate, and glycerol monostearate leads to the formation of three-dimensional networks capable of immobilizing vegetable oils without altering their chemical composition [49,50,51].

Organogel formation depends on a critical gelation concentration, while the crystallization onset temperature increases nonlinearly with the organogelator fraction, following a fractal behavior with an approximate dimension of 2.75. This fractal microstructure dictates the system’s rheological properties and, consequently, its release performance [49]. Rheological characterization further elucidates how gel network architecture governs molecular mobility and may modulate the oxidative substrate diffusion and release kinetics [50].

The release of active agents occurs through combined diffusion and carrier-erosion mechanisms, with erosion being the predominant mechanism in monoacylglycerol-based systems [51]. Modulating the ratio between gelators allows adjustment of structural density and partial control over the distribution of the released compound between the gastric and intestinal tracts. In this sense, the organogel microstructure serves as an engineering tool for regulating bioaccessibility and release kinetics [51].

At the mechanistic level, the gelation process begins with the dissolution of the oleogelator in the oil at elevated temperatures, followed by a cooling step that induces the self-assembly of the gelator molecules through noncovalent interactions, primarily hydrophobic interactions, van der Waals forces, and molecular packing of hydrocarbon chains. This process generates crystalline domains that grow into lamellae, fibers, or anisotropic crystals, which subsequently interconnect to form a three-dimensional network that immobilizes the liquid oil phase [95].

From a structural perspective, the encapsulating molecule (oleogelator) adopts a highly ordered organization in which hydrophobic chains align to form lamellar or fibrillar crystalline domains, as detected by techniques such as X-ray diffraction, polarized microscopy, and rheological analysis. These crystalline structures connect with one another, forming a percolating supramolecular network that physically traps oil within nanometric interstitial spaces, stabilizing the system against oil migration and oxidative degradation [96].

In this type of structured system, lipophilic bioactive molecules are not located within the gelator crystal lattice but instead remain dissolved in the oil phase, confined within the structural network. In this way, the oil acts as a solubilizing medium, while the crystalline gelator network functions as a physical matrix that limits molecular mobility and modulates the release of bioactive compounds. This mechanism enhances the chemical stability and bioavailability of lipophilic compounds, as the supramolecular framework can reduce oxygen diffusion, exposure to digestive enzymes, and oil oxidation [95,97].

In systems based on EVOO, the presence of phenolic compounds may also modify the supramolecular organization of the oleogel. These compounds have been reported to interact with certain gelators via hydrogen bonding or aromatic interactions, thereby generating additional junction points within the crystalline network and reinforcing the gel’s mechanical and thermal properties. Consequently, increasing phenolic content can alter crystal morphology and enhance the structural strength of the oleogelated system [98].

Taken together, these studies demonstrate that organogels based on vegetable oils function as hierarchical supramolecular matrices, in which the gelator’s molecular self-assembly produces crystalline networks that trap oil and stabilize lipophilic bioactive compounds. This makes them promising platforms for the preservation, protection, and controlled release of functional compounds in lipid-based matrices.

For EVOO applications, organogels can be framed as structural preservation systems; they limit oxygen diffusion and physical mobility, potentially improving stability while enabling functional product formats. These systems offer significant advantages, including reduced molecular mobility, potential limitation of oxygen diffusion, and preservation of the oil’s chemical identity. Furthermore, structural stabilization may facilitate integration with sensor-ready packaging technologies aimed at monitoring oxidation and authenticity, thereby reinforcing advanced quality assurance strategies.

However, organogels depend on critical gelation conditions and are sensitive to temperature fluctuations. Structural modification of EVOO may not always be desirable, and while oxygen diffusion can be reduced, oxidative reactions are not fully eliminated.

### 4.7. Comparative Synthesis of Nano-Strategies

Table 1 synthesizes representative nano-enabled systems, their target bioactives, reported benefits, and practical constraints. A recurrent theme is that translational readiness depends less on nanoscale novelty than on food-grade manufacturability, oxidative compatibility of excipients, validation in real oil matrices, and compatibility with EVOO category requirements.

**Figure 6:**
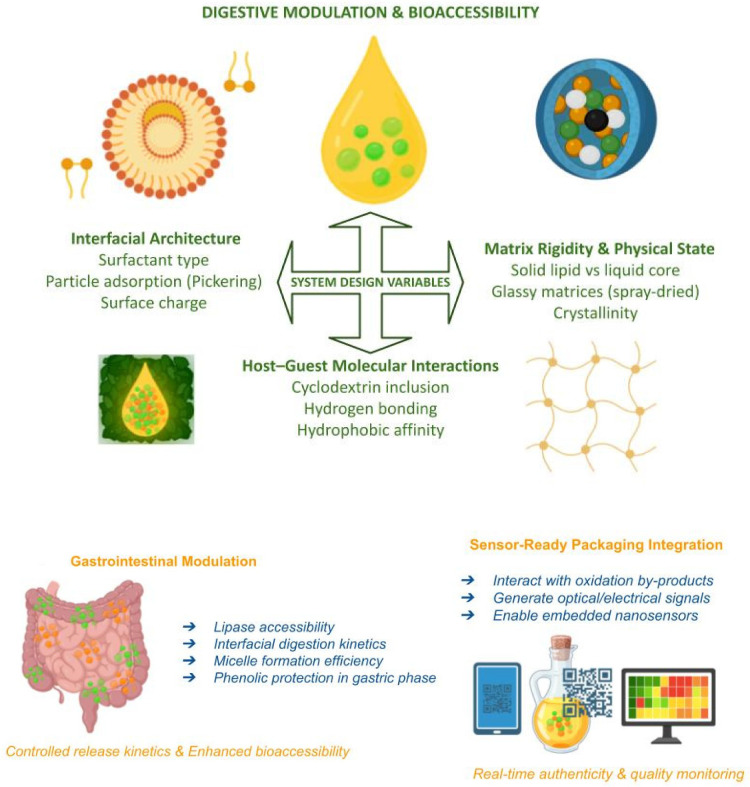
Nano-engineered routes linking stabilization determinants, gastrointestinal modulation, and smart authenticity workflows for EVOO-oriented systems.

Figure 6 summarizes the main nano-engineered routes that connect stabilization, digestion control, and smart authenticity assurance in EVOO-oriented systems.

## 5. Analytical and Physicochemical Characterization Workflows

A nano-enabled EVOO strategy is only as credible as its characterization pipeline. Two analytical objectives must be satisfied:

1. Carrier characterization (size, morphology, surface charge, encapsulation, stability).
2. Oil quality and bioactive integrity (phenolic profile, volatile markers, oxidation indices, sensory quality).

### 5.1. Characterization of Nanoformulations

For colloidal delivery systems, particle size distribution and polydispersity are typically assessed using DLS, with support from microscopy (TEM/SEM/AFM) and surface charge measurements (zeta potential). Encapsulation efficiency and release kinetics require compound-specific quantitation—most commonly LC-MS or HPLC-DAD for phenolics [13,14,21,22,23]. Thermal behavior (DSC), crystalline structure (XRD), and rheology (for gels/emulsions) provide a structural–functional foundation for interpreting stability [50,51].

### 5.2. Monitoring EVOO Quality and Oxidative Status

EVOO quality is conventionally assessed using peroxide value and UV absorption indices; however, modern workflows integrate chromatographic and spectroscopic measurements to link chemical changes to sensory outcomes. Volatile profiling by GC-MS and headspace techniques is critical for detecting rancidity markers and sensory defects [17,18,30]. Packaging and storage studies consistently show that such markers can track degradation trajectories and are sensitive to oxygen and light transmission [25,26,27,28,29].

### 5.3. Real-World Quality Control and Official Standards for EVOO

From a food-oriented translational perspective, EVOO quality management is ultimately constrained by the official category definition: extra virgin status is granted only when both chemical criteria and sensory panel requirements are met and maintained through the declared minimum durability date. The International Olive Council (IOC) trade standard and the European Union (EU) marketing standards harmonize this framework and specify the analytical methods used for conformity assessment [109,110,111].

In routine control, “what is measured” is therefore not arbitrary: free acidity (hydrolytic degradation), peroxide value (primary oxidation), and UV indices (K232/K270 and ΔK; conjugated dienes/trienes and refined-oil signatures) are interpreted together as orthogonal indicators of oxidation status and processing history. In parallel, fatty acid ethyl esters (FAEEs) and other regulated purity parameters provide additional safeguards against poor handling, soft deodorization, or blending practices that may escape a single test [109,110,111].

Beyond regulated criteria, industry laboratories increasingly deploy complementary freshness and processing markers—such as pyropheophytins (PPP) and 1,2-diacylglycerols (1,2-DAGs)—as risk-screening tools to flag aged, heated, or soft-deodorized oils for confirmatory testing. While these markers are not universally embedded in IOC/EU category limits, they are supported by a growing evidence base and are widely discussed in the authenticity literature [112,113].

Key official parameters, typical EVOO limits, and their interpretation for shelf-life and fraud control are summarized in Table 2.

### 5.4. In Vitro Digestion and Bioaccessibility

To evaluate whether nano-enabled preservation translates into functional delivery, standardized digestion models are increasingly used. The INFOGEST protocols provide consensus static and semi-dynamic in vitro digestion frameworks suitable for comparative evaluation across studies and formulations [60,61]. Their use enables quantitative assessment of phenolic release, micellarization, and matrix effects. This is particularly important for EVOO phenolics given their interactions with lipids, bile salts, and digestive enzymes, which can reshape effective bioaccessibility beyond mere chemical stability. Table 3 summarizes an integrated analytical toolbox for nano-enabled EVOO systems.

## 6. Authentication and Traceability: Nano-Enabled Sensing and Data Systems

### 6.1. Chemical Fingerprinting and Metabolomic Authenticity

NMR, LC-MS, and GC-MS remain the most informative laboratory-scale tools for authenticity confirmation because they provide high-specificity fingerprints and compound-level data [17,18,21,22,23,31,32]. Their strength is analytical resolution; their weakness is cost, instrument complexity, and limited suitability for in-line or field deployment.

### 6.2. Rapid Spectroscopy and Chemometrics

By contrast, FTIR/NIR/Raman platforms paired with chemometrics are better positioned for rapid screening at mills, bottling plants, or inspection points. These approaches are faster and less destructive, but their reliability depends heavily on calibration sets that capture variability in cultivar, season, packaging, and storage [29,30,31,32].

In practical QC terms, the two layers should not be conflated. High-resolution spectroscopy and chromatography are confirmatory or reference methods. Portable or simplified optical systems are screening tools that triage samples for further analysis. The most credible workflows combine both levels rather than replacing one with the other.

### 6.3. Nano-Enabled Sensors and Smart Packaging Concepts

Nano-enabled sensing can contribute to EVOO control in two complementary modes: (i) rapid, in situ screening of freshness or oxidation proxies and (ii) targeted detection of adulteration markers or atypical fingerprints. In practice, deployment requires explicit performance figures in real oil matrices and decision thresholds anchored to official QC criteria (Section 5.3).

Electrochemical sensors illustrate the translation challenge for phenolic markers: Bounegru and Apetrei developed a tyrosinase-based biosensor (carbon nanofiber-modified electrode) for hydroxytyrosol, achieving an LOD of 0.40 μM and LOQ of 1.21 μM, and enabling discrimination of EVOO extracts as a function of storage time [114].

Optical approaches coupled with chemometrics are closer to industrial deployment because they are non-destructive and high-throughput. For instance, fluorescence monitoring of olive oils has been shown to predict oxidation-related quality indices, with cross-validated r^2^ values of 0.90–0.94 across samples and storage conditions [115].

Similarly, FT-NIR, combined with robust variable selection (BOSS–PLS), quantified EVOO adulteration levels, with R^2^ ≈ 0.99 and low prediction errors (RMSEP ≈ 1.68%), supporting rapid screening prior to confirmatory analysis [116].

Multisensor platforms can also deliver practical sensitivity at relevant fraud levels: the BIONOTE multisensor system detected extraneous vegetable oils in EVOO at <5% and olive-pomace oil at ~8%, with high classification performance reported under controlled conditions [117].

Representative sensor cases, performance metrics, and validation contexts are summarized in Table 4. Across modalities, translation hinges on matrix-aware calibration, external validation, drift control, maintenance under routine use, and integration with official conformity-check workflows rather than stand-alone proof-of-concept demonstrations.

For real-time deployment, the critical questions are not only analytical sensitivity but also fouling, calibration drift, sample preparation burden, and the ability to link sensor signals to official parameters such as PV, K232, K270, ΔK, or validated adulteration thresholds. Many published sensor studies remain below this validation threshold.

### 6.4. Digital Traceability, Machine Learning, and Blockchain

Traceability in EVOO should be treated as an integration problem rather than as a single digital tool. Chemical fingerprints, sensor outputs, logistics records, and batch metadata must be merged into auditable decisions. Machine-learning models can assist this process, but only if they are trained on representative datasets and externally validated [71,72,73,74,75].

Blockchain may strengthen record integrity and multi-party auditability, but it does not validate the accuracy of the incoming data [76,77,78]. In other words, it can secure provenance claims only when analytical checkpoints and sampling procedures are already trustworthy.

A pragmatic implementation pathway within current EVOO QC frameworks is tiered. First, producers and control laboratories perform routine IOC/EU conformity checks. Second, portable spectroscopic or sensor platforms screen large numbers of batches at critical control points. Third, anomalous lots are escalated to confirmatory NMR, LC-MS, GC-MS, or targeted purity testing. Finally, validated results and logistics events are stored in interoperable digital records or blockchain layers for audit trails, recall readiness, and chain-of-custody verification.

**Figure 7:**
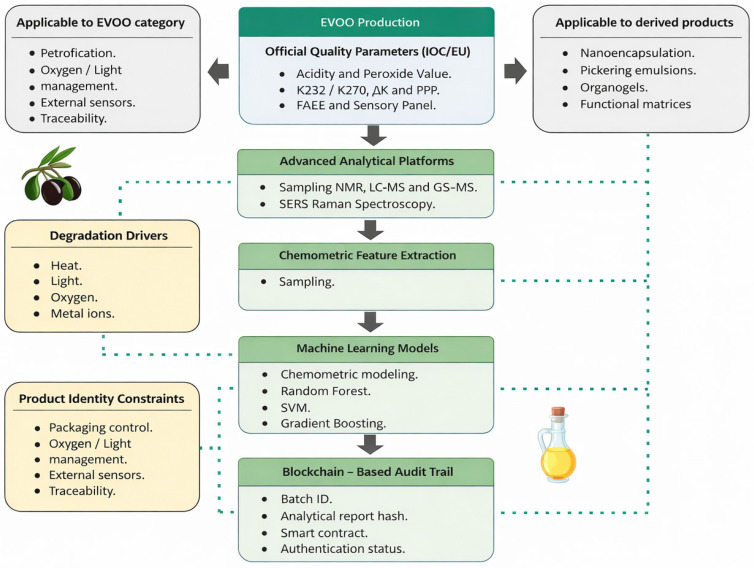
Nano-enabled smart traceability architecture for EVOO. Schematic integrating nano-enabled sensing at production/storage nodes, periodic chemical fingerprinting (NMR/LC-MS/GC-MS), machine learning classification models for anomaly detection, and blockchain-based audit trails for multi-party provenance, aligned with food fraud prevention frameworks.

Figure 7 illustrates a multi-layered control architecture designed to enhance transparency, authenticity verification, and fraud prevention across the EVOO value chain by combining advanced analytical chemistry, nano-enabled sensing, and digital decision systems. Rather than relying on a single analytical checkpoint, the model distributes verification across production, processing, and distribution stages.

At the upstream level, batches are characterized by chemical and spectroscopic fingerprints that capture phenolic composition, oxidation status, and compositional integrity.

These data are transmitted to centralized platforms, where machine-learning models compare each batch against curated reference libraries and assign anomaly scores rather than simple binary labels.

Validated outputs are then written into secure digital records so that regulators, producers, and certification bodies can audit decisions and trigger predefined actions for flagged lots.

This architecture does not replace conventional QC. It augments it by linking rapid screening, confirmatory analytics, and digital traceability into a single surveillance workflow.

## 7. Sustainability and Circular Economy: Integrating By-Product Valorization with Nano-Enabled Preservation

### 7.1. Circularity as a Value-Chain Redesign Challenge (Not an End-of-Pipe Fix)

Sustainability in the olive oil sector cannot be reduced to “waste treatment” alone. Across agri-food systems, conventional disposal routes for agricultural residues—such as uncontrolled burning, incineration, open dumping, or landfilling—are increasingly recognized as contributors to air pollution and broader ecological pressures, particularly in contexts of resource scarcity and rising demand for biomass-derived products [124,125]. Within the EVOO industry, the implementation of the circular economy (CE) should therefore be framed as a value chain redesign problem, in which residues become secondary raw materials and technology deployment is coupled with governance, markets, and verification tools.

Contemporary CE definitions converge on maintaining the utility and value of materials for as long as feasible through strategies such as reuse, recycling, and cascading valorization [126,127,128]. For olive oil supply chains, this conceptual lens is operationally relevant because the sector generates substantial residues from cultivation and harvesting (e.g., pruning biomass and leaves) and from milling and extraction (e.g., pomace and olive mill wastewater, OMW). At the EU scale, olive biomass residues have been estimated to amount to tens of millions of tons annually, underscoring both the environmental burden and the magnitude of the recoverable carbon and bioactive fractions [129]. Importantly, evidence from case-based and management-oriented analyses indicates that CE transition is frequently constrained by non-technical barriers (economic viability, regulatory uncertainty, market development, logistics), making business model innovation and stakeholder alignment central to CE feasibility in the olive sector [129,130].

From an implementation perspective, CE in EVOO should be understood as a multi-stakeholder architecture. Policy instruments can reshape incentives and compliance boundaries; industrial symbiosis can reduce transport and processing costs; and financing mechanisms can accelerate adoption of new valorization infrastructures (e.g., anaerobic digestion, biorefineries, nanomaterial production lines). In short, circularity requires integrating technical solutions with organizational models that capture value from residues, rather than treating them as liabilities [129,130,131].

### 7.2. Decision Intelligence for Circular Deployment: LCA-Driven Prioritization and Data-Enabled Optimization

A critical weakness in CE narratives is the implicit assumption that “circular” automatically means “more sustainable.” In practice, circular pathways can shift burdens upstream (e.g., energy demand, solvents, transport, or auxiliary chemicals) and therefore require quantitative assessment. Life Cycle Assessment (LCA) remains the most established framework for identifying environmental hotspots and comparing alternative waste management scenarios in EVOO systems [132,133].

Comparative LCAs in the olive sector consistently highlight the importance of both the agricultural phase and downstream packaging and logistics, while emphasizing that waste management choices can strongly influence overall impacts. For example, LCA comparisons between anaerobic digestion (AD) and conventional soil disposal of olive-derived residues indicate that AD can reduce environmental burdens by converting organic waste into biogas (offsetting fossil energy use) and by mitigating impacts associated with uncontrolled residue spreading [132]. At a higher level of synthesis, a systematic review specifically addressing CE principles within EVOO LCAs found that closed-loop strategies (where resources from by-product valorization are reintegrated into the production system) tend to yield more favorable environmental profiles than open-loop use cases, and identified agriculture and packaging as recurring hotspots [133].

Beyond classical LCA, CE execution is increasingly strengthened by data-driven tools. Process monitoring, data analytics, and AI-enabled optimization can improve residue logistics, predict variability in by-product composition, and guide the selection of extraction or conversion routes under real-world operational constraints [131]. However, methodological heterogeneity (system boundaries, functional units, allocation rules) remains a structural challenge for comparability and standardization, reinforcing the need for harmonized LCA/CE reporting frameworks tailored to olive supply chains [133].

### 7.3. Circular Bioactive Recovery as the Upstream Feedstock for Nano-Enabled Preservation

A circular strategy becomes technologically meaningful when it provides standardized functional inputs to high-value applications—precisely where nano-enabled preservation platforms can be applied. Olive residues (leaves, pomace, OMW) contain phenolic compounds and other specialized metabolites that can be recovered and positioned as antioxidant/functional ingredients, including for encapsulation, structured lipid systems, or active materials discussed earlier in this review.

Olive leaves are increasingly recognized as a significant reservoir of bioactive compounds, and compositional profiling studies emphasize their potential as sources of antioxidant and health-relevant compounds across cultivars and seasons [134,135]. From a separation standpoint, scalable fractionation and purification routes are essential to transition from “extracts” to specification-grade ingredients; in this context, membrane-based separations and related intensification platforms bridge recovery and industrial feasibility [136]. In parallel, advanced extraction comparisons on exhausted olive pomace demonstrate that intensified methods (e.g., ultrasound-assisted extraction and accelerated solvent extraction) can enhance the recovery of antioxidants and biological activity, strengthening the case for “first-step valorization” before energy use or further material conversion [137].

A distinct nano-enabled opportunity is the use of nanohybrid materials as stabilization matrices for recovered bioactives. For instance, incorporating polyphenol-rich olive leaf extracts into layered double hydroxide (LDH) architectures has been proposed as a strategy to enhance stability and functional performance, thereby aligning bioactive recovery with material engineering for downstream applications [138]. Importantly, such recovery-to-formulation pipelines must remain compatible with food and materials safety expectations, including guidance on nanomaterials and risk assessment requirements in regulated settings [82,83].

### 7.4. Waste-to-Nanomaterial Conversion: Nanocellulose and Biochar as Circular Functional Platforms

Circularity in EVOO is not limited to extracting molecules; it also includes converting lignocellulosic residues into functional solids that can substitute fossil-derived additives. Two residue-to-nanomaterial pathways stand out for their strategic relevance: (i) nanocellulose and (ii) biochar/nanobiochar.

First, olive pruning biomass and pomace-derived cellulose fractions can be processed into micro/nanofibrillated structures that act as reinforcing agents in biodegradable matrices and as functional stabilizers in dispersed systems [106,139,140,141]. These routes directly support nano-enabled preservation by enabling (a) more sustainable packaging architectures and (b) particle-stabilized interfaces (e.g., Pickering-type systems) where renewable fibers contribute to physical stability and barrier performance without relying on petrochemical surfactants [38,39,40,41,42,43,44,106,139].

Second, thermochemical conversion of olive residues into biochar yields carbon-rich materials whose properties depend strongly on the feedstock and processing conditions (e.g., pyrolysis, gasification, hydrothermal carbonization). A comprehensive review of production techniques and environmental applications highlights biochar’s relevance for adsorption and remediation, as well as emerging functional roles in catalytic and electrochemical contexts [142]. Olive mill solid waste-derived biochar has been specifically studied for heavy metal removal, illustrating a pathway in which “waste” can serve as a remediation material with measurable environmental benefits [143]. Moreover, recent syntheses emphasize that coupling biochar with microbial processes can further enhance heavy-metal removal, thereby indicating hybrid strategies for the management of contaminated water and soil [144]. At the agronomic interface, global-scale datasets on biochar’s effects on crop yields, soil properties, and greenhouse gas emissions support evidence-based deployment and contextualize where nano/biochar applications are likely to be most effective [145].

Collectively, these residue-to-nanomaterial pathways extend the CE logic beyond molecule recovery and enable cascaded, multi-sector use cases (food systems, agriculture, environmental remediation), which is often necessary to achieve economic viability at scale.

### 7.5. Sustainable Packaging as a Circular Lever at a Recurrent Hotspot

Packaging frequently appears as an LCA hotspot in EVOO supply chains, making it a prime target for circular innovation [133]. From a CE standpoint, the key objective is to deliver barrier and mechanical performance while reducing reliance on petrochemical plastics and improving end-of-life outcomes.

Olive by-product-derived nanocomposites provide a credible pathway. Cellulose nanofibers extracted from olive tree pruning have been proposed as additives in biodegradable films, improving functional performance relevant to food packaging [140]. Similarly, cellulose microfibers from olive pomace have been used to reinforce green composites, supporting sustainable packaging development while valorizing milling residues [141]. When integrated with the nano-enabled preservation strategies reviewed earlier (e.g., incorporation of antioxidant fractions or controlled-release architectures), these materials enable a “dual function” approach: packaging that is not only greener but also supports oxidative stability and shelf-life protection through improved barrier properties and active components.

### 7.6. Energy Recovery and Environmental Remediation: Closing Loops Without Shifting Burdens

Circular deployment in EVOO must also account for energy and wastewater challenges. Olive residues can be redirected toward energy generation (e.g., anaerobic digestion, co-digestion, thermochemical routes), but the sustainability of these pathways depends on conversion efficiencies, logistics, and local energy displacement factors.

Energy valorization studies and reviews describe multiple routes for converting olive-derived waste into bioenergy and highlight the need to select pathways compatible with the residue’s moisture content and regional infrastructure [146,147]. More broadly, lignocellulosic waste valorization frameworks (biofuels, biocomposites, bioplastics) provide a technical basis for integrating olive residues into diversified circular product portfolios [148]. For the valorization of pruning residues, energetic routes such as pyrolysis and gasification have been summarized, with attention to process options and outputs, thereby reinforcing the role of pruning as a non-trivial biomass feedstock [149]. Importantly, advanced system designs can go further: a multiproduct olive-pruning biorefinery coupled with carbon capture has been assessed as a potential net-negative pathway in specific configurations, illustrating how CE strategies can intersect with decarbonization objectives beyond simple waste reduction [150].

Wastewater streams require equally robust solutions. Photocatalytic approaches have been investigated for treating olive mill wastewater using activated-carbon-supported catalysts, demonstrating a strategy in which catalytic materials and process engineering work jointly to reduce pollutants [151]. Complementarily, biological valorization routes using oleaginous yeasts and bioprocesses can simultaneously detoxify wastewater and produce high-value metabolites, suggesting integrated “treatment-plus-product” models rather than remediation alone [152,153].

Table 5 summarizes the principal circular valorization routes, the resulting nano-enabled outputs, and their connections to EVOO preservation and sustainability targets.

### 7.7. Valorization of Olive Oil Industry Biomass Within a Food-Oriented Circular Biorefinery Framework

Beyond end-of-pipe management, olive-mill residues constitute a valuable resource for food systems, enabling the recovery of antioxidant phenolics and functional fiber fractions. Recent syntheses highlight scalable routes for valorizing olive mill wastewater (OMWW), pomace, and leaves into food-grade extracts and ingredients within a circular biorefinery logic [157,158,159].

Olive pomace and exhausted pomace can be upgraded via intensified extraction to produce phenolic-rich fractions with nutraceutical potential, while the residual lignocellulosic matrix can support downstream applications (e.g., functional fiber ingredients, materials, and energy) within integrated circular portfolios [137,148,158].

OMWW is a concentrated aqueous stream of polyphenols; membrane-based fractionation enables the concentration and purification of bioactives, supporting ingredient standardization and integrated detoxification/valorization [136,157,158]. When bioactive recovery is coupled to biological routes (e.g., oleaginous yeasts), the platform shifts from treatment to co-production of high-value metabolites [152,153].

Olive leaves are a well-established reservoir of oleuropein and related phenolics [9,11,134,135]. Food-format deployment is increasingly supported by encapsulation and structuring approaches (e.g., β-cyclodextrin complexes) and by micro- and nanoencapsulated extracts evaluated in bakery matrices and digestion models [37,47,48,60,61].

**Figure 8:**
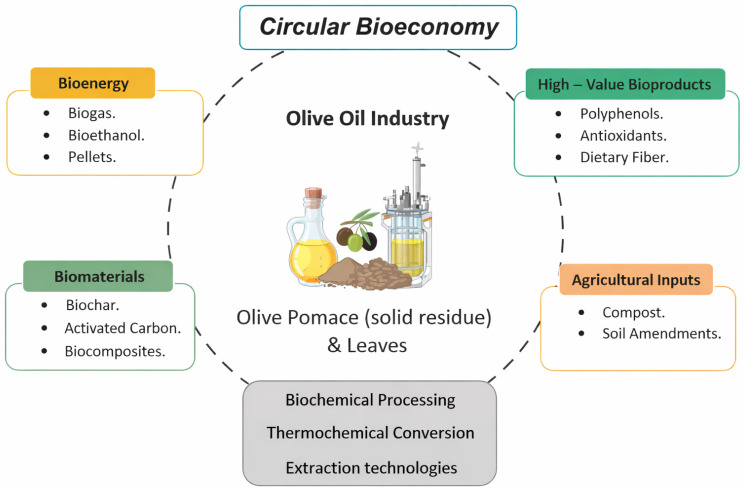
Circular bioeconomy pathways for olive-oil-industry biomass, showing how major by-products can be redirected toward bioactive recovery, biomaterials, energy, remediation, and agronomic return.

From a Food-oriented perspective, the priority is cascading valorization: (i) recovery and stabilization of phenolics for antioxidant and functional-ingredient applications, followed by (ii) conversion of remaining biomass to energy, remediation materials, or agronomic amendments, guided by circular economy principles and life-cycle thinking [129,130,131,132,133]. Figure 8 summarizes the principal product families and transformation routes, whereas Table 6 maps key biomass streams to food-relevant components, applications, and processing strategies.

Together, these food-oriented routes operationalize a circular biorefinery hierarchy in which ingredient recovery and stabilization precede downstream energy/material conversions, aligning valorization with both quality preservation and sustainability objectives.

## 8. Regulatory, Safety, and Consumer Acceptance Considerations

### 8.1. Regulatory Frameworks and EFSA Guidance

Regulation is a decisive constraint for food nanotechnology. EFSA guidance provides a structured approach to risk assessment of nanomaterials in the food and feed chain, emphasizing characterization, exposure assessment, toxicological evaluation, and uncertainty management [83]. EFSA technical requirements for establishing the presence of small particles, including nanoparticles, further underscore the importance of analytical demonstration and standardized measurement [82]. For EVOO applications, this implies that nano-enabled ingredients and packaging components must be accompanied by robust characterization (e.g., size distribution, dissolution, migration) and safety documentation appropriate for the intended use.

### 8.2. Safety-by-Design and Material Selection

A practical “safety-by-design” approach favors materials with established food use histories (e.g., phospholipids, proteins, polysaccharides) and production methods that avoid residual solvents and reactive contaminants. Systems such as cyclodextrin inclusion complexes and biopolymer-based structures can be strategically advantageous because they may reduce the novelty of regulatory interpretation [37,59]. Nonetheless, each application must address migration, stability under realistic storage, and interactions with the oil matrix.

### 8.3. Consumer Acceptance and Trust

Consumer acceptance is often underestimated in technically oriented research. In EVOO markets, authenticity is strongly linked to heritage, artisanal narratives, and perceived naturalness. Thus, nano-enabled interventions must be framed as quality protection and trust enhancement, not as “industrial alteration.” Transparent labeling, traceability, and evidence-based communication are essential, especially when nanotechnology terminology raises concerns.

### 8.4. Product Identity Constraints: EVOO Category Compliance vs. Derived Products

A central translational constraint is that many nano-enabled strategies are formulation changes rather than simple preservation tools. Under IOC and EU standards, EVOO must be obtained solely by mechanical or physical means and must meet chemical and sensory criteria. Additives are not permitted in virgin olive oils, and category compliance is verified through standardized conformity checks [107,108,109,110,111].

Therefore, strategies that can be applied while preserving the EVOO category are mainly external or non-formative: improved packaging, oxygen management during filling and storage, non-contact sensing, and digital chain-of-custody tools. These options can strengthen quality protection without changing the oil itself [110].

By contrast, nanoencapsulation, Pickering emulsions, cyclodextrin inclusion complexes, organogels, and phenolic enrichment are better framed as derived products or functional formulations. They may deliver stability or bioaccessibility gains, but they also create a distinct regulatory and labeling context [82,83,109,110,111].

## 9. Future Directions and Research Priorities

Several research and translational priorities emerge:

- Mechanism-grounded formulation design: More work is needed to connect carrier structure (interfacial architecture, rheology, crystallinity) to oxidation kinetics and phenolic retention in real packaging and storage conditions [24,25,26,27,28,29,53].
- Standardized performance metrics: Consistent reporting of encapsulation efficiency, release kinetics, and stability conditions is essential for cross-study comparisons. Routine adoption of standardized digestion methods (INFOGEST) should be standard when making functional delivery claims [60,61,62,63].
- Scale-up and manufacturability: Many nano-systems remain at the lab-scale proof of concept stage. Cost of goods, process reproducibility, and quality control strategies need to be integrated early [56,57,58,59].
- Integrated authenticity architectures: The future likely involves multi-layer verification—rapid spectroscopic screening combined with targeted confirmatory tests and digital traceability. ML models should be designed for auditability and to be resilient to confounders [31,32,71,72,73].
- Circular biorefinery integration: Valorization of by-products should be linked to high-value outputs like phenolic extracts, nanocellulose stabilizers, and bio-based packaging, creating a closed-loop innovation system [106,126,127,128,157,158,159].
- Regulation-ready innovation: Aligning early with EFSA guidance and technical requirements can prevent regulatory issues at later stages. Use of food-native materials and transparent risk assessments will be key.

Taken together, the most promising near-term directions are those that combine food-grade preservation with minimal disruption of EVOO identity: improved packaging and oxygen management, matrix-validated rapid screening linked to confirmatory analytics, and circular recovery of phenolics or packaging materials from olive side streams. The main gaps remain scale-up, interlaboratory validation, regulatory readiness, and demonstration in real commercial supply chains.

### Recent Evidence Reinforcing Translational Readiness

Recent literature reinforces this translational perspective. Updated reviews of olive-oil by-product valorization show that green extraction, encapsulation, and food applications can be integrated within circular biorefinery models [159]. In parallel, geographically resolved phenolic profiling continues to support the value of preserving minor EVOO compounds during processing and storage [160].

Recent storage studies further show that packaging design materially affects phenolic retention and sensory evolution, with higher-barrier systems improving preservation [161]. At the formulation level, current reviews on nutraceutical nanoencapsulation and sustainable nanoemulsification indicate that food-grade carriers can improve stability and controlled release, but only when the system is validated in realistic lipid matrices [162,163].

## 10. Conclusions

EVOO is simultaneously a premium nutritional lipid and a chemically delicate bioactive system. Its commercial success depends on preserving phenolic integrity and sensory quality while protecting against fraud in increasingly complex global supply chains. Nanotechnological strategies provide a powerful but non-trivial toolkit: lipid nanoparticles, polymeric carriers, cyclodextrin complexes, nanoemulsions, Pickering systems, and organogels can each contribute to stability, controlled release, and functional product innovation. Yet success is conditional on robust analytical characterization, digestion of relevant validation, and industrial realism. The most promising pathway is convergent; nano-enabled preservation integrated with authenticity sensing and digital traceability, underpinned by circular valorization of olive by-products and guided by regulation-ready safety frameworks. In this view, nanotechnology is not an isolated “tech layer” but a strategic enabler of a resilient, trusted, and sustainable EVOO value chain.

## Figures and Tables

**Table 1:** Representative nanotechnological strategies for EVOO/olive-derived bioactive preservation and delivery.

Platform	Target Bioactive/Matrix	Typical Materials	Key Benefit	Key Challenge	Representative Refs.
Nanostructured lipid carriers (NLCs)	Oleuropein/olive phenolics	Structured lipid blends (solid–liquid) + surfactant	High loading, protection, improved bioavailability, and potential controlled release	Carrier oxidation; surfactant selection; long-term physical stability; scale-up	[33,99,100]
Polymeric nanoparticles	Olive leaf phenolic extract	PLA and related biodegradable polymers	Robust structure; controlled release and stability	Food-grade processing; residual solvent/migration	[35,101,102]
Liposomes	Olive leaf polyphenols/oleuropein	Phospholipid bilayers	High compatibility; protection; aqueous dispersibility; improve bioaccessibility	Stability under processing, cost, and leakage	[36,103,104]
Cyclodextrin inclusion complexes	Olive leaf extract	β-cyclodextrin	Molecular encapsulation; improved stability; functional ingredient format	Complexation efficiency; sensory impacts	[37,105]
Nanoemulsions	EVOO or olive oils with phenolics	Food surfactants/emulsifiers	High dispersion stability; functional delivery	Surfactant safety and sensory; oxidation at interfaces	[45,46,58]
Nanocellulose Pickering emulsions	Olive oil/EVOO	Cellulose nanocrystals, proteins	Interfacial barrier; enhanced stability; renewable materials	Particle sourcing; rheology control; regulatory clarity	[38,39,40,41,42,43,44,106]
Hybrid nanoparticle stabilizers	Olive oil components/lipophilic bioactives	β-CD + polysaccharide nanoparticles	Improved storage stability via multi-mechanism protection; interfacial network formation	Formulation complexity; reproducibility; scale-up	[107]
Organogels	Olive oil structured systems	Policosanol, fatty alcohols, monoacylglycerols	Diffusion limitation; novel textures; delivery formats	Consumer acceptance; oxidation under long storage	[49,50,51]
Spray-drying microencapsulation	Olive oil (oil powder, rich in oleocanthal)	Proteins/polysaccharides (walls)	Oxidation protection; powder format; preservation	Reconstitution; process heat; wall selection	[52,108]
Food matrix incorporation	Biscuits/foods with phenolic carriers	Micro/nanoencapsulated extracts	Improved stability in foods; digestion-phase evaluation	Thermal processing: polyphenol–matrix interactions	[47,48]

**Table 2:** IOC/EU official quality criteria and complementary markers used in real-world EVOO quality control (QC), shelf-life management, and fraud risk screening.

Parameter/Marker	What It Measures (QC Target)	IOC/EU Status & Typical EVOO Limit	Typical Method	Interpretation for Shelf-Life & Fraud Control	Key Refs.
Free acidity (% as oleic acid)	Hydrolytic degradation (triacylglycerol hydrolysis)	Official criterion: ≤0.8% (EVOO)	Titration	Category compliance: Elevated values indicate poor fruit/storage. Not sufficient alone because refining/blending can reduce acidity.	[109,110,111]
Peroxide value (PV; meq O_2_/kg)	Primary oxidation (hydroperoxides)	Official criterion: ≤20 (EVOO)	Iodometric titration	Early oxidation and shelf-life risk. Must be interpreted in light of UV indices because PV may decrease after decomposition or deodorization.	[109,110,111]
K232	Conjugated dienes (primary oxidation)	Official criterion: ≤2.50 (EVOO)	UV spectrophotometry	Tracks early oxidation; supportive for shelf-life prediction and for identifying atypical processing histories.	[109,110,111]
K270	Conjugated trienes/secondary oxidation products	Official criterion: ≤0.22 (EVOO)	UV spectrophotometry	Sensitive to secondary oxidation and some refining/deodorization patterns; key for shelf-life and fraud screening.	[109,110,111]
ΔK	Spectral deviation (refining signature)	Official criterion: ≤0.01 (EVOO)	UV spectrophotometry	Designed to flag refined oil signals and certain adulteration patterns; complements K232/K270.	[109,110,111]
Sensory panel (Md, Mf)	Organoleptic defects and fruitiness	Official criterion; Md = 0.0 and Mf > 0.0 (EVOO)	IOC/EU sensory panel test	Detects defects and deodorization attempts that may evade chemistry-only control; critical for category integrity.	[109,110,111]
Fatty acid ethyl esters (FAEEs)	Fermentation/poor handling markers	Official criterion: ≤35 mg/kg (EVOO)	GC-based methods	Risk indicator for poor fruit quality and potential soft deodorization; used for targeted confirmation and supply-chain triage.	[109,110,111]
Purity profile (fatty acids, sterols, waxes, aliphatic alcohols, stigmastadienes, etc.)	Compositional authenticity markers	Official ranges/limits (category-dependent)	GC-FID/GC-MS (targeted)	Detects blending with seed oils, pomace oil, or refined oils; essential for fraud control beyond oxidation indices.	[110,111,112]
Supplementary freshness/processing markers (PPP; 1,2-DAGs)	Ageing/heat history; soft deodorization risk	Not universally embedded in the IOC/EU category limits	HPLC/GC; targeted markers	Used as risk-screening to flag aged/heat-treated/soft-deodorized oils for confirmatory tests; complements IOC/EU parameters.	[112,113]

**Table 3:** Analytical toolbox for nano-enabled EVOO preservation, delivery, and authenticity assessment.

Analytical Domain	Technique	Primary Readout	Application to EVOO Nano-Systems	Representative Refs.
Colloidal structure	DLS/nanoparticle tracking	Size, PDI	Carrier stability; aggregation risk	[56,57,58,59]
Surface properties	Zeta potential	Surface charge	Dispersion stability; interfacial behavior	[56,57,58,59]
Morphology	TEM/SEM/AFM	Shape, structure	Particle integrity; Pickering stabilizers	[38,39,40,41,42,43,44,106]
Thermal behavior	DSC/TGA	Phase transitions	Lipid carrier crystallinity; organogel structure	[49,50,51]
Rheology	Shear/oscillatory tests	Viscoelasticity	Organogels; Pickering networks	[50,51]
Phenolic profiling	HPLC-DAD/LC-MS/MS	Compound-specific concentration	Bioactive retention; release kinetics	[13,14,21,22,23]
Aroma and rancidity	GC-MS, headspace	Volatile markers	Shelf-life; oxidative fingerprint	[17,18,30]
Oxidation indices	PV, UV indices, Rancimat	Oxidative status	Packaging/storage comparisons; model calibration	[24,25,26,27,28,29,53]
Digestion models	INFOGEST static digestion	Bioaccessibility metrics	Release, micellarization, stability in GI	[60,61]
Rapid fingerprinting	NMR, FTIR/NIR/Raman	Spectral patterns	Authenticity screening + chemometrics	[31,32]
Antioxidant capacity assays	DPPH, ABTS (and related) radical-scavenging equivalents	Spectrophotometric radical-scavenging assays	Comparative screening of antioxidant preservation across formulations/extracts	[62,63]

**Table 4:** Representative sensor-based studies for EVOO quality and authenticity monitoring, with performance metrics reported in real oil matrices.

Sensor Modality	Target/Task	Matrix & Sample Prep	Performance Metrics	Validation/Interference Notes	Refs.
Electrochemical enzymatic biosensor (tyrosinase; carbon nanofibers)	Hydroxytyrosol as a freshness/phenolic marker	Phenolic extract from EVOO (hydroalcoholic extraction)	LOD 0.40 μM; LOQ 1.21 μM	Selectivity governed by phenolic oxidation; requires an extraction step; calibration against storage-dependent changes; matrix complexity and co-extracted compounds may interfere; robustness depends on enzymatic stability and validation against reference methods	[114,118,119]
Fluorescence spectroscopy + chemometrics	Oxidation monitoring (PV, UV indices proxies)	Direct oil measurement (non-destructive)	Cross-validated r^2^ ≈ 0.90–0.94 (model-dependent); RMSE comparable to reference titrimetric/UV methods	Matrix fluorescence is sensitive to temperature/light history; inner-filter effects and pigment overlap; requires multivariate calibration and drift control	[115,120,121]
FT-NIR spectroscopy + BOSS–PLS	Adulteration quantification (mixtures)	Direct oil spectra (rapid screening)	R^2^ ≈ 0.992; RMSEP ≈ 1.68% (adulteration level)	Sensitive to cultivar/harvest variability; suitable as a screen prior to confirmatory targeted tests; requires calibration	[32,116]
Multisensor platform (BIONOTE: e-nose + e-tongue)	Detection of extraneous oils/pomace oil	Headspace + liquid measurements (platform-specific)	Detects extraneous vegetable oils <5%; pomace oil ~8%	Performance depends on the training set and environmental control; best used with routine calibration and reference samples	[117,122,123]

**Table 5:** Circular valorization routes in the olive oil sector and nano-enabled outputs relevant to EVOO preservation and sustainability.

Residue Stream (Olive Sector)	Circular Valorization Route	Nano-Enabled Output (or Enabling Material)	How It Connects to Nano-Preservation and/or Sustainability Targets	Key Refs.
Olive leaves	Polyphenol recovery; profiling for cultivar/seasonal robustness	Polyphenol-rich extracts; nanohybrid stabilization matrices (e.g., LDH-based)	Supplies standardized antioxidant inputs for encapsulation/active materials; improves stability of recovered phenolics	[134,135,138]
Exhausted olive pomace	Advanced extraction intensification (UAE/ASE/MAE comparisons)	High-activity antioxidant extracts (e.g., hydroxytyrosol-rich fractions)	“First-step valorization” that feeds high-value applications before energy/material conversion; supports cascading use	[137,154]
Pruning biomass/pomace fibers	Cellulose extraction → microfibers/nanofibers	Nanocellulose-reinforced biodegradable films	Improves barrier/mechanical performance in green packaging; reduces reliance on petro-plastics (packaging hotspot)	[106,139,140,141]
Pomace/pruning residues	Thermochemical conversion (pyrolysis/gasification/HTC)	Biochar/nanobiochar functional solids	Adsorbents/catalyst supports for remediation; supports circular remediation materials and soil applications	[142,143,144,145]
Olive mill wastewater (OMW)	Microbial valorization	Single-cell oils, carotenoids, organic acids + detoxification	Converts a high-load waste stream into products; reduces environmental burden via co-production	[152,153]
OMW	Photocatalytic treatment	Supported photocatalysts (e.g., TiO_2_-based composites)	Wastewater remediation with engineered catalysts complements biological valorization in integrated treatment trains	[151,155]
Pruning biomass	Multiproduct biorefinery + CCS/BECCS (scenario-dependent)	Decarbonization-aligned bioenergy/bioproduct system	Potential net-negative emissions configurations; connect CE with climate mitigation targets	[150,156]

**Table 6:** Food-oriented valorization of biomass streams from the olive oil industry.

Biomass Stream	Key Compounds/ Components	Main Food Applications	Extraction/Processing Strategies	References
Olive Pomace (incl. exhausted pomace)	Phenolic fractions; dietary fiber; residual lipids	Polyphenol-rich extracts; fiber-enriched foods; nutraceutical ingredients	Advanced/green extraction; fractionation and stabilization; milling/drying for fiber incorporation	[137,148,158,159]
Olive Mill Wastewater (OMWW)	Soluble polyphenols; organic acids	Natural antioxidants; functional ingredient concentrates (post-purification)	Membrane fractionation; adsorption/green extraction; coupled bioprocessing for co-products	[136,152,153,154,155,156,157,158]
Olive Leaves	Oleuropein; flavonoids; secoiridoids	Functional ingredients; natural preservatives; encapsulated bioactives for food matrices	Hydroalcoholic extraction; inclusion complexes; micro-/nanoencapsulation; drying	[9,11,37,47,48,134,135]
Olive Stones (Pits)	Lignocellulosic matrix	Indirect food-system sustainability (bioenergy); filtration/catalyst-support materials	Pelletization; thermochemical conversion; activated-carbon-based materials	[149,151]
Composted/Soil-Returned Residues	Stabilized organic matter	Soil amendment supporting sustainable olive cultivation and circular nutrient cycling	Controlled composting; LCA-informed management selection	[132,133]

## Data Availability

No new data were created or analyzed in this study. Data sharing is not applicable to this article.
